# Exploring the Feasibility and Acceptability of a Brief Online Dialogic Book-Sharing Training for Teaching Support Staff

**DOI:** 10.3390/children11121423

**Published:** 2024-11-26

**Authors:** Judy Hutchings, Rebecca Lothian, Anwen Jones, Margiad E. Williams

**Affiliations:** Centre for Evidence Based Early Intervention, Bangor University, Bangor LL57 2DG, UK; j.hutchings@bangor.ac.uk (J.H.); rbl22qcx@bangor.ac.uk (R.L.); anwen.r.jones@bangor.ac.uk (A.J.)

**Keywords:** school support staff, training, school intervention, book-sharing, children, language skill deficits, expressive language, social–emotional competence, feasibility

## Abstract

Background/Objectives: In the UK, significant and rising numbers of children arrive in schools with marked deficits in key skills such as oral language. This rise has been further negatively impacted by the COVID-19 pandemic. Given this, the foundation phase of primary school education is a necessary environment for targeting language deficits. There is evidence to suggest that teaching assistant (TA)-led interventions can be effective when adequate training and support are provided. This study explored the feasibility of providing a brief, online dialogic book-sharing training to TAs, and whether this training would be effective in upskilling TAs and enable them to improve the language outcomes of children aged 3–7 years in a school context. Methods: North Wales primary schools were invited to nominate TAs for the two half-day training sessions. Five schools responded, and eleven TA–child dyads participated. Data were collected on recruitment, training acceptability and baseline, and post-training measures from TAs and children (2–3 weeks after the final training session) and 4–6 weeks after the first follow-up. Measures of TA competence and behavior were collected, along with measures of child language and behavior. Results: Schools and TAs were recruited; TAs reported positively to the training, and the results showed small to large effect size benefits on all TA skills and child expressive language with significant positive post-training effects on TAs’ use of reflections and child language abilities. However, these effects were somewhat reduced at follow-up. Conclusions: Overall, the results of this feasibility study provide positive evidence for this training as an accessible way for schools to strengthen their prevention infrastructures by professionalizing a growing, but relatively untrained, group within the school workforce.

## 1. Introduction

Increasing numbers of children, particularly those from low socioeconomic backgrounds, are entering school without the necessary levels of understanding and expression to be able to access the curriculum [1]. This is a public health concern because these difficulties typically persist and determine both academic and economic achievement at later stages in life [1]. Children’s speech, language, and communication needs, across health and education settings, are often not identified [2]. This problem worsened due to the impact of the COVID-19 pandemic lockdowns. In the UK, COVID-19 recovery plans were developed for schools to support children struggling with both speaking and understanding [3].

Dialogic book-sharing (DBS) is an evidence-based intervention, originally designed to optimize parental use of picture books, which can substantially improve young children’s language development [4]. DBS encourages adults to make several changes to how they typically share books with children, the most significant change being a shift in roles. Typically, the adult plays an active role by reading the words in a book verbatim, and the child passively listens. However, with DBS, adults become active listeners and encourage children to assume a progressively more active role in storytelling. Adults support this by following the child’s lead, sensitively responding to their interests, and encouraging them to actively participate in conversations by increasing both the number and complexity of questions asked and with the provision of maximally informative feedback to the child [5]. This benefits both preschool and school-aged children’s expressive language and has been successfully implemented by parents and early years educators [6,7].

DBS could be a promising school-based intervention for children entering school with additional language and communication needs; however, to date, no research has been conducted in the UK with school-based support staff delivering DBS to children. Recent US studies have delivered DBS training to teaching assistants (TAs) who were supporting their schools’ preschool programs [8,9]. Fleury and Schwartz [8] found that TAs could be trained to effectively incorporate DBS into their daily practice, with high fidelity; when they did, children with autism spectrum disorder, regardless of severity, learned new book-specific vocabulary more efficiently, remained engaged with book-sharing activities for longer, and increased their rate of verbal responses to TA comments and questions about the book. However, Towson et al. [9] found that TAs implemented DBS strategies with variable fidelity following a single 45 min training session and with additional support provided by scripted books. Post-intervention, three of the four TAs significantly changed the way they shared books by increasing their use of CROWD (Completion, Recall, Open-ended, Wh-, Distancing) prompts. The training also provided TAs with knowledge of the evaluate, expand, and repeat strategies, but their use of these skills was variable and inconsistent and did not reach a level of significance. Also, when the scripted book support was removed, TAs could not develop their own prompts. Despite these conflicting results, overall, these studies suggest that TAs may be a viable workforce for expanding the use of this promising method with young children arriving at school with language delays.

In Wales, the Foundation Phase [10] and subsequent Curriculum for Wales were introduced to support children as active participants in the learning process [10]. Play and first-hand experiences are prioritized, and practitioners promote adult–child interactions that involve sustained shared thinking, open-ended questions, and reflexive co-construction of knowledge [10,11]. To meet higher adult-to-child ratios (1:8, particularly in nursery and reception classes (3–5-year-olds)) required to facilitate these developments, additional TAs have been recruited [12].

### Study Aims

Given the increasing numbers of children entering school with speech and language deficits [13], and that these children are more likely to receive TA support [14,15], TAs could potentially benefit from training to use evidence-based DBS skills. This was a feasibility study to trial a brief, online DBS training for TAs currently supporting children aged 3–7 years. The main aim of the trial was to assess the feasibility of recruitment and delivery of the training and to explore the acceptability of the training and whether there were observed post-training changes in TA behavior and in children’s language skills.

## 2. Materials and Methods

### 2.1. Design

North Wales primary schools were invited to nominate TAs for a DBS training programed to enhance children’s language skills. Pre- and post-training data were collected from TAs and children between May and July 2023. This study reports on recruitment, acceptability of the brief online dialogic book-sharing training, and the impact on TAs’ and children’s outcomes using a repeated measures design via questionnaires, direct observation of TA–child book-sharing interactions, and a gaming-format-based child language assessment.

### 2.2. Procedures

#### 2.2.1. Recruitment

Study details were sent via email to North Wales primary schools. Eleven schools expressed interest. Once ethical approval was granted, schools were sent another email requesting that they provide TAs with notes of interest and return the completed notes to the researcher to enable the researcher to obtain formal consent and arrange the baseline school visit. Schools were asked to identify children whom they believed would benefit from a language intervention and to contact their parents, using a pre-written email provided by the researcher, for consent for their child’s participation. The email included a link to a Microsoft Form that contained an information sheet and consent form for parents. Parental consent included approval for their child to be filmed with the TA during a 10 min book-sharing activity on three separate occasions.

Five schools responded; two were small rural schools (pupil numbers 42 and 99), and one was a rural school with 198 pupils. Two were large schools in urban areas (pupil numbers 410 and 435). Twelve TAs were recruited. One TA and child dyad was withdrawn as the TA was absent due to illness during the baseline assessment and the first training session and one child was absent at baseline data collection.

#### 2.2.2. Training Development

The Books Together program [7] was adapted to create a brief online dialogic book-sharing training for TAs. Books Together is a seven-session group-based parent intervention that has demonstrated benefits to child language development when delivered to parents via schools in Wales [7]. The current study involved two three-hour online training sessions, delivered one week apart via Zoom. Session 1 covered the themes of weeks 1–3 and session 2, the themes of weeks 4–7 (see Table 1). A Books Together trainer delivered the content live using a PowerPoint presentation. Training included video examples of parents demonstrating good book-sharing practices with their child, activities that used the interactive whiteboard function on Zoom, and breakout room practice sessions in which TAs practiced the skills with each other.

The core training principles were identical to those taught in the Books Together program [7] with the exception that no coaching of TAs with children during the training was possible. TAs were taught skills to support children’s interest and active engagement rather than focusing on reading the text. They were encouraged to respond flexibly and sensitively to children’s developmental capacity and experience. The importance of positive reinforcement, through praise and reflecting back and expanding children’s verbalizations, was emphasized.

### 2.3. Measures

#### 2.3.1. Acceptability and Feasibility Outcomes

Participants completed an end-of-training evaluation questionnaire. A link to the online form was provided in the Chat function on Zoom and via email to the headteachers. Topics covered included the usefulness of the training overall and specific aspects of the training including the video clips, practice sessions, discussions, and summary sheets. Items were rated on a five-point Likert scale ranging from 1 = strongly disagree to 5 = strongly agree. The questionnaire also asked about whether participants would prefer a Welsh language version of the training.

#### 2.3.2. Demographic Questionnaire

The basic demographic information included TA and child age and gender and TA employment status, education level, and experience working with children generally and with the child in this study.

#### 2.3.3. Child Behavior

The Teacher Strengths and Difficulties Questionnaire (T-SDQ) [16] is a screening tool for assessing child behavior. It has a total problem score comprising four problem behavior subscales (conduct, emotional, hyperactivity, peer problems) and a prosocial behavior subscale. The English language version for children aged 4–17 years was used. The SDQ includes 25 items—for example, “Generally obedient, usually does what adults request”—rated on a three-point Likert scale rated not true, somewhat true, and certainly true. Higher problem scale scores indicate greater levels of difficulties. This measure was used at baseline only to provide a sample description.

#### 2.3.4. Teaching Assistant Sense of Competence

Adapted from the Parental Sense of Competence Scale [17], this 17-item scale measures satisfaction with, and efficacy in, participant roles as a TA. An example is “Working with children sometimes makes me tense and anxious”. Each item is rated on a six-point Likert scale, ranging from 1 = strongly disagree to 6 = strongly agree. Nine items are reverse-coded. The sum of scores for all items is the total score. Higher scores indicate a stronger sense of competency. Surface adaptations included changing the word parent to TA.

#### 2.3.5. TA Behaviors

The Dyadic Parent–child Interaction Coding System (DPICS) [18] is a well-established observation tool that measures the quality of adult–child interactions. In the current study, it was used to measure TA–child interactions during a 10 min book-sharing activity. Observations were video recorded for later coding to allow assessment of inter-rater reliability. Ten behaviors were coded including both positive (praise, encouragement, reflections, questions, verbal labeling, verbal questioning, emotion coaching, and linking), and negative behaviors (critical statements and response opportunity [whether TAs provided children opportunity to respond]). Coding sheets recorded the frequency of each behavior by scoring a mark in the applicable tally box each time the behavior occurred.

#### 2.3.6. Child Language

The Early Years Toolbox [19], an iPad-based game, normed for children aged 3–5 years, that asks children to name cartoon images of objects (e.g., flower, vegetables), was used to assess children’s expressive language. Responses were recorded by the researcher on the iPad app, using one of three options: correct response, other response, or don’t know. The measure includes 55 items, and administration takes an average duration of 5 min. Stop rules end the game after six consecutive incorrect and/or don’t know responses. Scores are calculated by summing the number of correct responses.

The 10 min TA–child video recorded observations during book-sharing were transcribed using four measures, single word utterances, multiword utterances involving two or more words, mean length of child utterances, and the total number of times that the child spoke. In accordance with Deshmukh and colleagues’ [20] methodology, any one-word-long utterance or an article and one word (e.g., “Yes”, “A chicken”) was categorized as a single word/basic utterance, including utterances with false starts (e.g., “The, the, the duck”). Two-or-more-word utterances were coded multi-word utterances (e.g., Red and yellow). The mean length of child utterance counted each string of words, and total utterance counted the number of times the child spoke during the 10 min observation.

### 2.4. Data Collection

TAs were contacted via telephone to arrange baseline school visits at a time that was convenient for them. Data were collected from participants during three school visits. A baseline assessment occurred once parental consent had been obtained, and TAs had read the participant information sheet and signed the consent form. The post-training assessment took place within three weeks of completing the training. The follow-up assessment occurred one month later.

At the baseline school visit, the researcher and the TA went to a quiet room alone. The researcher reminded the TA what their participation in this study involved, informed them of their participant number and their right to withdraw at any time, and obtained written informed consent. TAs then completed the brief demographic questionnaire, SOC, and T-SDQ. The T-SDQ was collected at baseline only to provide demographic information. Training packs comprising seven books and two booklets containing summaries and examples of the skills from each training session were provided (see Table 1).

Once the questionnaires were completed, TAs collected the children from their classrooms. The researcher introduced themselves to the child and explained that they would be helping their TA by first playing a quick game on the iPad (i.e., the Early Years Toolbox—delivered by the researcher with the TA present to ensure the child felt comfortable). The TA and child were then video recorded whilst sharing a book. The camera was set up on a tripod stand in front of the table at which the TA and child were sat side by side. The researcher provided books from the Usborne Farmyard Tales series, counterbalanced across the three data points to reduce practice effects. The TA was instructed to look at the book with the child for 10 min. For the five TA–child dyads from the Welsh medium school, the Welsh versions of the same books were provided. A second book from the same series was provided for all TAs to ensure that they could fill 10 min of observation.

All video-recorded TA–child observations were transcribed and coded by the first author (primary coder). The criterion coder (last author) coded 25% of randomly selected videos for inter-rater reliability (IRR). Interclass correlation (ICC) estimates and their 95% confidence intervals were calculated based on a single-rating, consistency, 2-way mixed-effects model. Researchers achieved excellent inter-rater reliability (between 0.932 and 1.000) across all scales.

### 2.5. Data Analysis

Measures of TA sense of competence, child behavior, language and length of utterance, and TA–child interactions were scored according to the guidelines for each measure. Descriptive statistics (means and standard deviations) were calculated. As this was a feasibility trial, with a small sample and range of measures, there was inadequate statistical power to detect effects ([21]), so results are presented in terms of effect size changes. Effect sizes are reported as Cohen’s *d*. Cohen [21] suggests the following categorization of effect sizes for *d*: 0.2 (small), 0.5 (medium), and 0.8 (large).

## 3. Results

### 3.1. Sample Characteristics

Twelve TA–child dyads were recruited from five primary schools across North Wales. Due to the absence of one TA and one child, from two different dyads within the same school at baseline data collection, a new dyad was formed using the TA and a child that was present in order to collect data from the TA who was present. Unfortunately, it was not feasible for the new dyad to practice DBS together as they were in different classes, and the child who was absent at baseline was used at the other two time points. Therefore, one TA–child dyad was withdrawn, and no child data from the other TA–child dyad were reported, resulting in data on 11 TAs and 10 children being reported.

The TAs were aged between 28 and 60 years old. Six stated English (55%) and three Welsh (27%) as their first language, and two (18%) identified as bilingual (English- and Welsh-speaking). All were paid employees at the schools. The majority worked full-time as general classroom TAs (82%), and two worked part-time in a resource provision unit with children with special educational needs (18%). Six TAs (55%) had obtained GCSEs. The others had no qualifications (*n* = 1, 9%), AS level (*n* = 1, 9%), or further education of A-level equivalent or above (*n* = 3, 27%). Experience working with children ranged from 3 to 30 years.

Children were aged between 4 and 7 years. Five children (50%) spoke English as their first/main language, four spoke Welsh (40%), and one was bilingual (10%). The bilingual child spoke English and Gujarati. Seven children knew their TA well (70%). The length of time that TAs had supported the children in this study ranged from 0 to 48 months. No behavioral issues were reported for the children, with 90% scoring close to average on the T-SDQ. Only one child (10%) had a slightly raised score. See Table 2 for further details.

Howard and Melhuish [19] only report EYT norms for children aged 3–5 years. All children completed the EYT, but at baseline, only seven children were within the 3–5-year age range. Of these, three were performing at or below the 25th percentile, one child at the 50th percentile, and three at the 75th percentile on the EYT [19]. The other three children were aged 6–7, and therefore, no score was calculated.

### 3.2. Training Engagement

All 11 TAs attended both training sessions and completed the program with their child partner. The majority joined training sessions from a quiet room at school, with one joining from home for the second session.

### 3.3. Acceptability of the Training

TA feedback on the training evaluation questionnaire was positive. The majority reported that all components were useful or very useful. One participant felt there was a need for Welsh medium training. These findings indicate good satisfaction levels (see Table 3).

### 3.4. Pre- and Post-Training Results

Baseline and two sets of follow-up measures were collected from all TA–child dyads (100%).

#### 3.4.1. TA Outcomes

Several TAs were already using many DBS skills in their regular practice, but there was large variation between individuals (see Table 4 and Table 5). Emotion coaching and linking book content to children’s own experiences were the least used behaviors at baseline (7.64 and 3.36 occurrences, respectively). There was an increase in frequency from baseline to the first follow-up and then a generally small decrease in frequency at the second follow-up for most observed outcomes, although they mostly remained at a higher level than at baseline. The exceptions were emotion coaching, which increased at each follow-up; praise, which decreased at each follow-up; verbal labeling, which decreased at the first follow-up and then increased at the second follow-up; and critical statements, which already occurred at a low frequency, stayed the same at the first follow-up, and increased slightly at the second follow-up. The greatest gains were found for linking, which almost tripled in post-training frequency (3.36 to 9.82 occurrences), and reflections and encouragement which both doubled post-training (13.27 to 29.00 and 7.73 to 15.64 occurrences respectively). Reflections remained close to double the baseline frequency at the second follow-up (13.27 to 25.91 occurrences).

Differences between baseline and post-training scores showed large effect size increases in reflections, linking, and verbal questions (large effect sizes; *d* = −1.87, −1.56, and −1.10, respectively), and comparisons between baseline and follow-up showed large effect size increases in reflections but a decrease in TAs’ sense of competence (large effect sizes; *d* = −1.51 and 0.82, respectively). The decrease in sense of competence was also shown in the comparisons between post-training and follow-up (medium effect size; *d* = 0.63).

#### 3.4.2. Child Outcomes

All children performing within the 75th percentile for the EYT at baseline maintained that level of functioning throughout. The child operating within the 50th percentile achieved a 75th percentile level of performance post-training. Two of the three children performing at or below the 25th percentile made improvements—one improved post-training, maintained at follow-up, and the other at follow-up.

In the post hoc comparisons, increases in children’s expressive language were found between baseline and post-training assessments (small to very large effect sizes; *d* = −0.25 to −2.95) and from baseline to follow-up (medium to very large effect sizes; *d* = −0.44 to −2.43), including for EYT, MWU, total number of utterances, and number of words (see Table 4 and Table 5).

## 4. Discussion

Increasing numbers of children are arriving at school with speech and language delays, and early years classrooms are now supported by growing numbers of teaching assistants. These staff are generally the least trained members of school support for children and express the need for additional training as they spend much of their time with children in need of additional support [15]. This study assessed the feasibility of recruitment of schools, TAs, and children and the acceptability of a brief online dialogic book-sharing training for TAs in Welsh primary schools. It also explored its impact on TAs’ use of DBS skills in supporting children’s language development, their sense of competence and whether any observed TA use of the skills impacted children’s language skills.

### 4.1. Feasibility Findings

Five North Wales primary schools were recruited and nominated 12 TAs, of whom 11 were present at the training and provided baseline and follow-up measures. Twelve parents agreed to their child taking part, including consent to their child being videotaped, and 10 children provided baseline and follow-up measures for the trial. TAs reported satisfaction with the training. These results suggest that the trial was feasible to deliver and that there was a perceived benefit to schools in enrolling for the trial.

### 4.2. Impact on TA Outcomes

The training changed TAs’ practice, with six of the eight positive behaviors increasing in post-training frequency with large effect size benefits. Overall, the mean effect size change of 0.93 post-training across the ten observed TA outcomes (Cohen’s *d* ranged from 0.36 to 1.87). This is in line with Dowdall et al.’s [6] meta-analysis for caregiver behavior changes. Effect sizes offer fewer misleading interpretations of training effects as they are not confounded by sample size [22]. The changes in behavior were maintained at the second follow-up, although the effect sizes were slightly diminished, with a mean of 0.88 at follow-up. Reflections were the most frequently used tool, in line with previous findings [7]. Verbal questions and linking were also used more frequently than other techniques.

TAs reported a reduced post-intervention sense of competence (SOC) at the second follow-up despite being satisfied with the training and still using the skills, although at slightly reduced rates, something that a future study could explore further. The significant reduction in TAs’ sense of competence contrasts with previous research with parents [7]. Whilst there was no effect of the training on TA self-reported competence between baseline and post-training, medium- and large-effect-sized reductions were observed between the two follow-ups and between the baseline and second follow-up, respectively. This could reflect more general levels of satisfaction in the TA role, which might naturally fluctuate with demands throughout the school year, rather than a specific effect of the training on their sense of competence in supporting children with their language development. Previous research has reported that TAs are dissatisfied with the lack of training and development opportunities available to them [15], and the training was well received with positive reports from the end-of-training evaluation questionnaire.

Most prior research does not measure changes in teacher behavior during book-sharing. Only Hargrave and Senechal [23] assessed teachers in terms of their questions or requests and the feedback they gave children (including praise, modeling correct answers, repeating child utterances, expanding child utterances). Some of these categories overlap with those in the current study, including verbal questions (wh-questions), praise, and reflections (repeating child utterances). Teachers in Hargrave and Senechal’s [23] study were seven times more likely to repeat children’s utterances (compared to 1.6 times in the current study) six times more likely to use wh-questions (compared to 1.6 times in the current study), five times more likely to use praise (compared to 0.9 times less likely in the current study) post-intervention. This suggests that providing TAs with DBS training benefits their use of book-sharing skills; however, the amount of training time might need to be increased, and boosters or direct skill coaching may be required to improve longer-term effects.

It is difficult to draw direct comparisons between studies regarding changes in adult behavior during book-sharing because studies have different foci that impact how they categorize the behaviors of interest. However, the large positive post-intervention effects of linking contrast with Williams et al. [7], who found small effects when the program was delivered to parents. For emotion coaching, the large positive effect in the current study was consistent with those found by Williams et al. [7] for social/emotional coaching and by Murray and colleagues [24] for mental state talk (which included references to cognitions, feelings, and desires).

Previous research has focused on DBS training for parents [7,23] and preschool teachers [25] with longer training; however, TAs did implement DBS skills with shorter training. However, the effects faded slightly over the month between the two post-training follow-ups, suggesting that more support is needed and highlighting the need for longer-term follow-up. Some TAs implemented the skills with better fidelity than others, as demonstrated by the large standard deviations. This may also reflect differences in the frequency of book-sharing sessions that TAs achieved, which can have a significant influence on outcomes [26].

### 4.3. Impact on Child Outcomes

Importantly, the intervention improved the number and length of children’s utterances. The current study adds support to growing evidence that DBS interventions improve children’s expressive language [6,27,28,29]. They also extend previous findings from preschool children by demonstrating efficacy with older school-aged children [8,23,25,26,30,31]. This has important implications for schools’ ability to meet the needs of the increasing numbers of children entering school with school readiness skill deficits, particularly in expressive language [13,32]. Improvements in language use could be explained by typical language development that occurs over time [33], and this could only be established with a control group. However, the time span was short, and the effect sizes were consistent with those found at post-intervention [23] and at follow-up [24] by others who used control groups or conducted a meta-analysis [6]. Additionally, the post-training assessment was conducted within the same time frame as that used by Hargrave and Senechal [23].

The findings from the naturalistic measures of language suggest a direct effect of the training as children produced a greater number of utterances, words, and longer sentences (i.e., MWU) immediately post-training when TAs’ use of the skills was at its peak, in line with previous findings [25,26]. In the current study, the follow-up data suggested a positive relationship between DBS behaviors and children’s language because the benefits for children reduced slightly at the second follow-up, coinciding with the reduction in TAs’ skill use at the second follow-up.

Overall, the results suggest that TAs can be trained to interact with children in ways that support child language development and have a positive effect on their academic progress [34]. The changes in child linguistic development coincided with the increased use of verbal questions and emotion coaching. Both involved asking more cognitively challenging questions, which required more independent thinking and speculation from the child [35]. Furthermore, increased use of encouragement and reflections, and minimal use of corrections (critical statements), encouraged children to construct their own understanding of events in the book [35,36,37,38]. This style of interaction is typically more prevalent in teacher–pupil interactions than TA–pupil interactions [14].

### 4.4. Limitations

This was a feasibility study, undertaken as a Masters by Research project by the first author. This limited the number of TAs that were recruited and trained. The sample was small (*n* = 11 TAs and *N* = 10 children), although this allowed for the feasibility questions to be explored. Child gender was female in 80% of the sample, and further trials involving more male children are required to determine whether developmental sex differences influence the impact of TA-led DBS interventions with this age group (3–7 years old). However, despite promising findings for both TAs and children evidenced in the reported effect size changes, the small numbers remain a significant limitation.

The training duration (two 3 h sessions) was less than that in Williams et al.’s [7] parenting trial (seven two-hour sessions). Additionally, because of the online training delivery format, TAs did not have the opportunity to be observed and coached in practicing the skills. The training length or lack of coaching may both have contributed to the fading of reported effects by the second follow-up. To overcome these limitations, future studies could explore whether a longer training with possibly some live supervision would be acceptable or feasible for schools. It would also be important to test the intervention with other ethnically diverse populations and in different geographic areas.

This study did not include a control group or directly measure the interaction between the changes in TAs’ behaviors and child language outcomes. Therefore, we cannot be certain that changes in children’s vocabulary acquisition occurred as a direct result of the DBS training. However, DBS encourages TAs to ask more complex, open-ended questions, encouraging children’s independent thinking and active participation [14,20,39]. This may have contributed to the significant increases in children’s number and length of utterances within such a short time frame.

Schools were asked to self-select for the project. This may have caused a selection bias whereby schools with stronger TA programs were more likely to sign up for the project. Given the very small sample size, and the risk of selection bias, it is vitally important that future research recruit larger samples from more diverse schools to minimize bias and increase the generalizability of the findings.

Although schools were asked to recruit children with speech and language needs, the trial did not impose inclusion criteria, despite suggesting that more attention to the recruitment of children might be beneficial. However, only three of the seven 3–5-year-old children were performing below age-expected levels. This suggests that further work needs to be undertaken with schools to ensure that those children most in need of support are recruited. Additionally, the tool for assessing children’s vocabulary, the EYT, was only normed for 3–5-year-old children, so these data were only available for seven (70%) of the children. A future study should identify a vocabulary tool that covers the age range of recruited children.

## 5. Conclusions

The COVID-19 pandemic resulted in an increase in the number of children arriving at school with language deficits, so evidence-based professional development training for school-based support staff, who are supporting many of these children, is needed. The current study provides preliminary evidence of the acceptability and efficacy of a brief online dialogic book-sharing training for teaching support staff, suggesting that TAs can improve child outcomes that are of public health importance, therefore providing justification for a larger, more rigorous randomized controlled trial with a specific focus on children from low-income backgrounds and with language delay.

## Figures and Tables

**Table 1:** Content of the dialogic book-sharing training for teaching support staff.

Training Day	Session Name	Corresponding Book
Day 1	Session 1: Introduction, Building and Enriching.	‘*Handa’s Surprise*’ by Eileen Browne
	Session 2: Linking	‘*Little Helpers*’ by Lynne Murray and Peter Cooper
	Session 3: Numbers and Comparisons.	‘*Handa’s Hen*’ by Eileen Browne
Day 2	Session 4: Talking about feelings.	‘*Hug*’ by Jez Alborough
	Session 5: Talking about intentions.	‘*Harry the Dirty Dog*’ by Gene Zimmerman
	Session 6: Talking about perspectives.	‘*Harry by the Sea*’ by Gene Zimmerman
	Session 7: Talking about relationships, Summary and Next Steps.	‘*All’s Well That Ends Well*’ by Lynne Murray and Peter Cooper

**Table 2:** Sample characteristics at baseline.

**TA Demographics**	**All (*N* = 11)**
TA age, years: *M* (*SD*)	49.91 (10.08)
TA gender, female: *n* (%)	11 (100.00)
Experience working with children, years: *M* (*SD*)	13.36 (8.41)
Length of time working with particular child, months: *M* (*SD*)	9 (13.40)
**Child Demographics**	**All (*N* = 10)**
Child age, years: *M* (*SD*)	5.36 (0.92)
Child gender, female: *n* (%)	8 (80.00)

**Table 3:** TA training evaluation responses.

Item	Modal Rating	Mean ± SD (Range)
1. I found the training beneficial	Agree	3.82 ± 0.60 (3–5)
2. The examples shown in the video clips were useful	Agree	4.00 ± 0.45 (3–5)
3. The practice sessions were useful	Agree	3.82 ± 0.60 (3–5)
4. The discussions after practice sessions were useful	Agree	4.00 ± 0.45 (3–5)
5. The summary sheets were useful	Agree	4.20 ± 0.42 (4–5)

Note. Scores ranged from 1 = strongly disagree, 2 = disagree, 3 = neutral, 4 = agree, 5 = strongly agree.

**Table 4:** Descriptives for child and TA outcomes.

Measure	Baseline *M* (*SD*)	Post-Training *M* (*SD*)	Follow-Up *M* (*SD*)
Child outcomes			
EYT	30.50 (10.41)	33.10 (11.05)	35.10 (11.04)
MLU	2.03 (1.01)	2.65 (1.23)	2.59 (0.95)
SWU	20.90 (11.69)	36.90 (15.96)	31.50 (14.33)
MWU	11.40 (9.22)	31.90 (21.33)	30.60 (15.54)
Total utterance	32.30 (16.45)	68.70 (25.47)	63.90 (23.35)
No. of words	64.10 (42.79)	190.50 (137.91)	168.20 (85.01)
TA outcomes			
SOC	67.36 (6.12)	66.18 (5.67)	62.36 (4.34)
Questions ^a^	20.64 (4.34)	26.45 (12.68)	26.82 (9.56)
Praise ^a^	8.55 (7.05)	7.91 (6.55)	5.09 (4.25)
Encouragement ^a^	7.73 (5.61)	15.64 (14.12)	14.18 (8.87)
Verbal labeling ^a^	14.27 (7.07)	11.72 (9.72)	12.18 (8.54)
Verbal questions ^a^	28.27 (15.26)	45.00 (16.93)	39.64 (18.10)
Reflections ^a^	13.27 (8.39)	29.00 (15.30)	25.91 (12.81)
Emotion coaching ^a^	7.64 (5.01)	12.64 (8.24)	13.55 (5.50)
Linking ^a^	3.36 (4.13)	9.82 (6.15)	5.82 (4.14)
Critical statement ^a^	0.09 (0.30)	0.09 (0.30)	0.45 (0.69)
No opportunity ^a^	13.82 (9.05)	18.73 (10.39)	15.82 (11.82)

Note. EYT = Early Years Toolbox; MLU = mean length of utterance; SWU = single-word utterance; MWU = multi-word utterance. ^a^ Observed variables.

**Table 5:** Effect size results for child and TA outcomes.

Measure	BL to Post *d*	Post to FU *d*	BL to FU *d*
Child outcomes			
EYT	−0.25	−0.19	−0.44
MLU	−0.62	0.06	−0.56
SWU	−1.37	0.46	−0.91
MWU	−2.22	0.14	−2.08
Total utterance	−2.21	0.29	−1.92
No. of words	−2.95	0.52	−2.43
TA outcomes			
SOC	0.19	0.63	0.82
Questions ^a^	−1.34	−0.08	−1.42
Praise ^a^	0.09	0.40	0.49
Encouragement ^a^	−1.41	0.26	−1.15
Verbal labeling ^a^	0.36	−0.06	0.26
Verbal questions ^a^	−1.10	0.35	−0.74
Reflections ^a^	−1.87	0.37	−1.51
Emotion coaching ^a^	−1.00	−0.18	−1.18
Linking ^a^	−1.56	0.97	−0.59
Critical statement ^a^	0.00	−1.21	−1.21
No opportunity ^a^	−0.54	0.32	−0.22

Note. BL = baseline; Post = post-training; FU = follow-up; EYT = Early Years Toolbox; MLU = mean length of utterance; SWU = single-word utterance; MWU = multi-word utterance. ^a^ Observed variables.

## Data Availability

All data generated for this study are available from the corresponding author upon reasonable request. The data are not publicly available due to ethical restrictions.
